# Metabolomic Analysis of Clinical Plasma from Cerebral Infarction Patients Presenting with Blood Stasis

**DOI:** 10.1155/2015/453423

**Published:** 2015-03-05

**Authors:** Min Ho Cha, Min Jung Kim, Jeeyoun Jung, Jin Hee Kim, Myeong Soo Lee, Myung-Sunny Kim

**Affiliations:** ^1^Medical Research Division, Korean Institute of Oriental Medicine, 1672 Yuseongdae-ro, Yuseong-gu, Daejeon 305-811, Republic of Korea; ^2^Research Group of Nutrition and Metabolic Systems, Korean Food Research Institute, 62 Anyangpangyo-ro, 1201 Beon-gil, Bundang-gu, Seongnam, Gyeonggi-do 463-746, Republic of Korea; ^3^Department of Food Biotechnology, University of Science & Technology, 62 Anyangpangyo-ro, 1201 Beon-gil, Bundang-gu, Seongnam, Gyeonggi-do 463-746, Republic of Korea

## Abstract

Blood stasis (BS) is characterized as a disorder of blood circulation. In traditional Korean medicine (TKM), it is viewed as a cause factor of diseases such as multiple sclerosis and stroke. This study investigated differences in the plasma metabolites profiles of subjects displaying BS or non-BS patterns. Thirty-one patients with cerebral infarction diagnosed with BS and an equal number of sex- and age-matched non-BS patients were enrolled. Metabolic profiling was performed using UPLC-MS. The ratio of subjects with a rough pulse and purple coloration of the tongue was higher in patients presenting with BS pattern. Through metabolomics analysis, 82 metabolites that differed significantly between the BS and non-BS pattern were identified, and the two groups were significantly separated using an orthogonal partial least square-discriminant analysis model (*P* < 0.001). Of these 82 metabolites, acetyl carnitine, leucine, kynurenine, phosphocholine, hexanoyl carnitine, and decanoyl carnitine were present in significantly higher levels in patients with a BS pattern than those with a non-BS pattern. Our results also demonstrated that seven plasma metabolites, including acyl-carnitines and kynurenine, were associated with a BS pattern, suggesting that variant plasma metabolic profiles may serve as a biomarker for diagnosis of BS in patients with cerebral infarction.

## 1. Introduction

Traditional Korean medicine (TKM) assigns diseases to subtypes according to the combination of symptoms experienced by patients, a process known as pattern identification (PI). PI is a diagnosis system unique to traditional medicines practiced in East Asian countries, including China, Korea, and Japan. In TKM, stroke is assigned to five PI subtypes: Qi-deficiency (QD), dampness-phlegm (DP), blood stasis (BS), Yin-deficiency (YD), and fire and heat (FH) [1].

Blood stasis (BS) is characterized as a disorder of blood circulation with hallmarks including extravagated or sluggish blood circulation and viscous or congested blood, all of which may contribute to various disease pathologies [1]. Similar to its definition in TKM, BS is described in traditional Chinese medicine (TCM) as a slowing or pooling of the blood caused by disruption of heart Qi. BS can be understood in biomedical terms in the context of hematological disorders such as hemorrhage, congestion, thrombosis, and local ischemia (microclots) [2].

The diagnosis of a BS pattern by traditional medical doctors relies on the symptoms exhibited by patients. The subjectivity of this process calls the reliability of a BS diagnosis into question [3], leading many researchers in China, Korea, and Japan to search for serum or plasma biomarkers associated with the diagnosis [4–7].

Metabolomics is a powerful approach to quantitative assessment of endogenous small molecules within biological fluids such as serum and urine [8] and has been utilized to identify candidate biomarkers of several diseases [9–13]. Many studies using this approach have been performed to find metabolites associated with PI subtypes [14–19]. Jian et al. have shown that some free fatty acids and amino acids, including octadecanoic acid, arachidonic acid, and proline, were associated with BS in coronary heart disease [18]. Zhao et al. have reported 37 metabolites associated with BS in patients with unstable angina [19].

In this study, we analyzed the metabolic profiles of plasma from patients with cerebral infarction (CI) by ultraperformance liquid chromatography-quadrupole time of-flight mass spectrometry (UPLC-Q-TOF-MS) to identify metabolites associated with BS.

## 2. Materials and Methods

### 2.1. Subjects

Patients with CI were enrolled from 2009 to 2010 at two Korean oriental medical hospitals (Kyung Hee Oriental Medical Center in Seoul and Dae Jeon Oriental Medical Hospital in Daejeon) and one western medical center (Dongguk University Medical Center in Kyunggi-do). Subjects were CI patients enrolled within one month of the onset of symptoms, as characterized by Park et al. [20]. The patients' symptoms were confirmed via diagnostic imaging with computerized tomography (CT) or magnetic resonance imaging (MRI). CI subtypes were determined according to the Trial of ORG 10172 in the Acute Stroke Treatment (TOAST) classification. Subjects with hemorrhaging or with infectious or liver diseases were excluded from the study. Some patient in this study was also included in a previous study [15].

After obtaining written informed consent from all subjects, clinical data and plasma were collected. This study was approved by the Institutional Review Board (IRB) of the KIOM and by both of the oriental medical hospitals.

### 2.2. Diagnosis of Blood Stasis Pattern

The symptoms experienced by subjects were collected using the “stroke PI case report form.” Diagnosis of a BS pattern in subjects was determined by two expert TKM doctors based on criteria established in “Korean Standard PIs for Stroke-II” as previously reported by Go et al. [21]. Subjects receiving different BS pattern diagnoses from the two doctors were excluded. There were 31 age- and sex-matched patients in the non-BS and BS groups. The general characteristics of patients in the two groups are shown in Table 1.

### 2.3. Plasma Extract Analysis by UPLC-Q-TOF-MS

Plasma protein was precipitated by the addition of cold acetonitrile. After shaking for 30 min at 4°C, the samples were centrifuged at 13,000 rpm for 10 min at 4°C. The supernatant was dissolved in 20% aqueous methanol containing caffeine for ultraperformance liquid chromatography-quadrupole time-of-flight mass spectrometry (UPLC-Q-TOF-MS) analysis.

A UPLC-Q-TOF-MS instrument (Waters, Milford, MA, USA), equipped with a column oven and coupled with a Waters Q-TOF primier, was used. The Q-TOF-MS was operated in positive electrospray ionization (ESI) mode with a scan range of* m/z* 50–1,000. Cone voltage was 30 V, capillary voltage was 3 kV, and scan time was 0.2 s with an interscan delay of 0.02 s. The source temperature was set at 110°C, and the desolvation flow was set at 700 L/h. The desolvation gas temperature was set at 300°C. The MS was calibrated using sodium format, and leucine enkephalin was used as lock mass. The concentration of leucine enkephalin was 200 *ρ*M, and the flow rate was set at 5 *μ*L/min.

In the MS-MS experiments, argon was used as collision gas, with the collision energy alternating between 10 and 30 eV. A 5 *μ*L aliquot of extracted plasma sample was injected into an Acquity UPLC BEH C18 column (2.1 × 100 mm, 1.7 *μ*m, Waters, Milford, MA, USA). The column oven was set at 40°C, and the sample temperature was 10°C. The mobile phase consisted of 0.1% formic acid in water (A) and 0.1% formic acid in acetonitrile (B), and the flow rate was 0.35 mL/min. MassLynx software version 4.1 (Waters Inc.) was used to control the instrument and calculate accurate masses.

### 2.4. Data Processing and Pattern Recognition Analysis

UPLC-MS data, including retention time,* m/z*, and ion intensity, were extracted using MarkerLynx software (Waters Corp., Milford, USA) and assembled into a data matrix. Peaks were collected using a peak width at 5% height, 1 s, a noise elimination of 6, and an intensity threshold of 50. Data were aligned with a mass tolerance of 0.07 Da and a retention time window of 0.2 min. All spectra were aligned and normalized to an external standard.

The resulting data sets were imported into SIMCA-P version 12.0.1 (Umetrics, Umeå, Sweden) for multivariate analysis and were mean-centered scaled. Orthogonal partial least-squares discriminant analysis (OPLS-DA) was conducted, and the quality of each model was determined based on a goodness of fit parameter (*R* ^2^) and a goodness of prediction parameter (*Q* ^2^). In addition, OPLS-DA models and the reliabilities of models were further validated using a rigorous permutation test (*n* = 200). To find metabolites that contributed to the discrimination, the S-plot showing a combination of covariance *p*(1) and correlation *p*(corr) from the OPLS-DA model was generated to better visualize the metabolites contributing to the discrimination. Assignment of metabolites contributing to the observed variance was performed using the ChemSpider (http://www.chemspider.com/) and the Human Metabolome Database (http://www.hmdb.ca/).

### 2.5. Statistical Analysis

The statistical analysis of our data was performed with IBM SPSS Statistics 19 (IBM Co., New York, NC, USA). After testing for the normality of continuous variables in clinical data by the Kolmogorov-Smirnov test, significant difference was determined via *t*-test for parametric variables or Mann-Whitney *U* test for nonparametric variables. Categorical variables were compared with a chi-square test or Fisher's exact test. The results of LC-MS were tested using independent *t*-test with Mann-Whitney *U* test. The statistical significance was set at *P* < 0.05.

## 3. Results 

### 3.1. Distribution of Symptoms and Signs in BS and Non-BS Groups

According to Korean Standard PIs for Stroke-II, we investigated the distribution of 11 diagnostic symptoms and signs of BS from patients with stroke. As shown in Table 2, 40.9% of the BS patients displayed purple tongue coloration (*P* = 0.001), and some patients in the BS group had red or black spots on the surface of the tongue which were not observed in the non-BS group (*P* = 0.039). In addition, the ratio of subjects with rough pulse in the BS group was significantly higher (64.5%) than that of subjects in the non-BS group (6.5%) (*P* < 0.001).

### 3.2. Pattern Recognition Analysis of Plasma Metabolic Profiling

**Figure 1:**
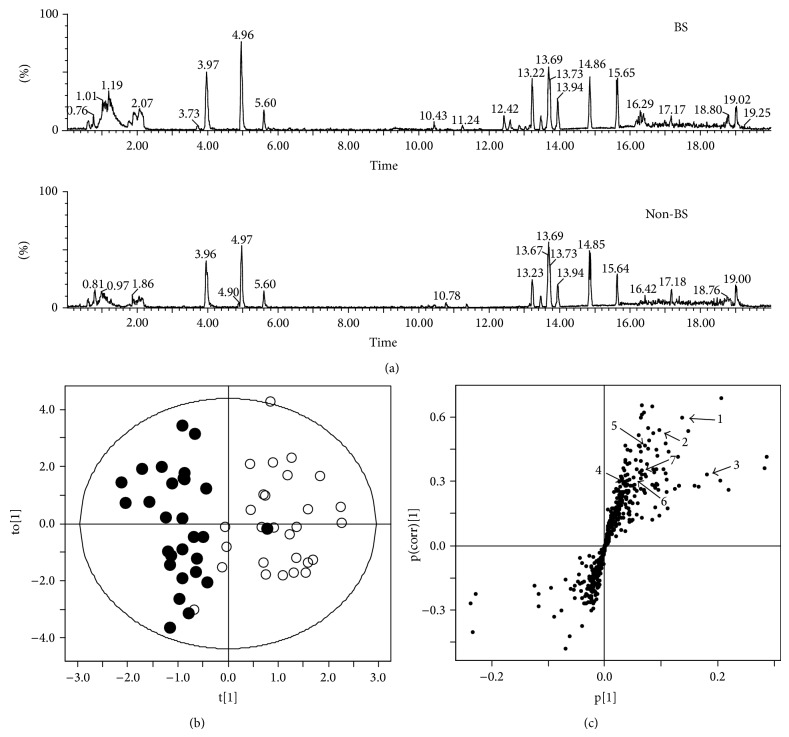
Ultraperformance liquid chromatography-quadrupole time-of-flight mass spectrometry (UPLC-Q-TOF-MS) profiles of plasma from BS and non-BS subjects in positive mode (a), orthogonal partial least-squares discriminant analysis (OPLS-DA) score (b), and S-plots derived from UPLC-Q-TOF MS data (c) of plasma from patients with stroke. The score plots showed a significant separation between BS and non-BS patients (*P* < 0.0001) by permutation test.

Figure 1(a) shows representative Base peak intensity (BPI) of plasma from the BS (higher) and non-BS patients (lower) obtained via UPLC-Q-TOF MS in positive mode. To identify differences in metabolic pattern between the BS and non-BS patients, OPLS-DA was applied to the UPLC-Q-TOF MS data.

The OPLS-DA score plot showed different metabolic patterns in the plasma of the BS and non-BS patients, and the *R* ^2^ *Y* and *Q* ^2^ *Y* values of the OPLS-DA model were 0.715 and 0.436, respectively (Figure 1(b)). The model was validated using 200x repeated permutation and with *P* < 0.0001, indicating that the OPLS-DA model is significant.

### 3.3. Targeted Metabolic Profiling to Identify a BS Marker

The UPLC-MS data allowed us to find 82 metabolites that differed significantly between the BS and non-BS groups (*P* < 0.05) in Supplemental Table 1 (see Supplementary Material available online at http://dx.doi.org/10.1155/2015/453423). Furthermore, we identified seven metabolites for which *P* < 0.05 and the variable importance of projection (VIP) value was higher than 1.0 using public data masses by ion masses determined using positive-ion-modes (Table 3).

Levels of carnitine-related metabolites, including acetyl carnitine, decanoyl carnitine, and hexanoyl carnitine, were significantly higher in the BS pattern than the non-BS pattern patients (*P* < 0.05). Kynurenine, which is produced by oxidation of tryptophan, was increased 2.2-fold in the BS pattern patients (*P* = 0.010). Creatinine, leucine, and phosphocholine were also increased 1.3–1.6-fold in the BS pattern patients (*P* = 0.010, *P* = 0.042, and *P* < 0.001, resp.).

## 4. Discussion

BS is defined as a state of stagnated or extravagated blood in a blood vessel that causes hematological disorders such as hemorrhage, congestion, thrombosis, and local ischemia (microclots) as well as changes in tissue [22]. In TCM, the major symptoms of BS pattern include purple coloration or the emergence of purple spots on the tongue. Additionally, BS often presents with a choppy or rough pulse [23]. The description of BS symptoms is similar in TKM [24], although the subjects in our study showed more exaggerated signs of purple color on tongue and rough pulse (Table 2). Inspection of the tongue holds a prominent place in TKM diagnostics, as various pathological conditions rooted in disruption of circulation manifest there [25, 26]. In particular, when the tongue is dark purple, stasis or hypoxia is present, rendering the microvenules dark blue [27]. Previous study reported that fibrinogen and platelet in plasma, which are parameters involved in blood coagulation, were increased in patients with BS [28], but those levels were not different in BS pattern (Table 1) and the syndrome of blood stasis in this study. However, it is unknown how these physiological changes occur and how they affect BS pattern.

Many studies have been performed in China, Korea, and Japan to find biological molecules affecting the BS patterns of various diseases [5, 17, 29]. Metabolomics is a powerful method for identifying small molecules present at different levels in the biofluids of patients affected by disease. Studies have been performed to relate BS patterns, as defined by TCM, with physiologically defined coronary heart disease, diabetes, and psoriasis [18, 19, 30]. In this study, we investigated plasma metabolites in BS and non-BS groups of patients with cerebral infarction using UPLC-Q-TOF-MS methods and found that the two groups were significantly separated in an OPLS-DA model obtained by plasma metabolic profiling (Figure 1(b)).

Several of metabolites, acetyl carnitine, hexanoyl carnitine, and decanoyl carnitine were significantly increased in the patients with a BS pattern (Table 3). Particularly, decanoyl carnitine and hexanoyl carnitine were related with tongue purple in color (data not shown). Carnitine also showed a tendency to increase in the patients with a BS pattern, in agreement with a previous study that elucidated BS of myocardial ischemia using TCM methods [31]. In addition, some studies also showed that the hypoxia-induced accumulation of long-chain acylcarnitine and repeated episodes of ischemia may cause chronic accumulation of short-chain acylcarnitine in plasma in patients with peripheral vascular disease [32].

Another metabolite, kynurenine, which is an intermediate in the tryptophan metabolic pathway producing niacin, was increased over twofold in the patients with a BS pattern (Table 3). Kynurenine is synthesized by tryptophan dioxygenase and indoleamine 2,3-dioxygenase cascades [33] and correlated with cardiovascular disease and stroke [34, 35]. The role of kynurenine in the BS pattern is unknown, but the relationship between kynurenine and BS pattern has been suggested by previous studies. One cause of BS pattern from a pathophysiological perspective is stagnant blood in a blood vessel, a phenotypic result of multiple sclerosis. Plasma kynurenine and indoleamine 2,3-dioxygenase activity have been known to increase in patients with atherosclerosis and coronary heart disease [36, 37]. Recently, Sulo et al. showed that the kynurenine-tryptophan ratio was an important predictor of coronary events, including multiple sclerosis [38]. We also observed that the kynurenine/tryptophan ratio in the BS subjects was increased. This means that the BS pattern caused by coagulation may activate the kynurenine pathway.

This study has several limitations. First, it is a cross-sectional study and, as such, does not demonstrate a change in plasma metabolite composition after treatment. Second, the sample size was small and may prevent generalizing the association of metabolites such as acylcarnitine and kynurenine with a BS pattern. Third, the degree of BS pattern in each of the patients was not quantified in this study; so, we could not determine whether a correlation between degree of BS and level of plasma metabolite biomarkers was present. Fourth, PI diagnosis was dependent on two expert TKM doctors, and the subjectivity of this diagnosis forced us to perform an observational study. Despite these limitations, this study provided metabolic information about BS patterns of stroke, and further studies should be performed in subjects of other large populations to generalize its conclusions.

## Supplementary Material

Suppl. Table 1: Identification of plasma metabolites in stroke patients (Non-BS and BS) analyzed using ultra-performance liquid chromatography-quadrupole time-of-flight mass spectrometry.

## Figures and Tables

**Table 1:** Demographic parameters of study subjects.

Characteristics	Non-BS	BS	*P* value^a^
Anthropometric characteristics			
Sex (M/F)	14/17	15/16	1.000^*^
Age (year)	67.13 ± 10.51	66.06 ± 10.78	0.659
Smoking (none/stop/active)	9/7/15	5/9/17	0.468
Drinking (none/stop/active)	9/5/17	17/3/11	0.120
BMI (kg/m^2^)	24.03 ± 4.23	22.85 ± 2.79	0.208
Waist circumference (cm)	84.10 ± 11.18	88.73 ± 9.69	0.209
TOAST classification			
LAA/CE/SVO/SUE	7/3/17/3	8/7/11/3	0.511
NIHSS	3.83 ± 3.09	3.53 ± 3.47	0.732
Medical history			
TIA (yes, %)	1 (3.23)	3 (9.68)	0.612^*^
Hypertension (yes, %)	19 (61.29)	20 (64.52)	1.000^*^
Hyperlipidemia (yes, %)	2 (6.45)	7 (22.58)	0.147^*^
DM (yes, %)	6 (19.35)	9 (29.03)	0.554^*^
IHD (yes, %)	0	3 (9.68)	0.238^*^
Blood parameter			
WBC (×10^3^)	7.93 ± 1.98	8.12 ± 2.43	0.734
RBC (×10^6^)	4.58 ± 0.48	4.44 ± 0.59	0.306
Hg (g/dL)	13.94 ± 1.60	13.60 ± 1.77	0.441
Hct (%)	40.10 ± 7.84	40.20 ± 4.75	0.949
Platelet (×10^3^/ul)	294.93 ± 98.24	284.27 ± 151.11	0.750
Fibrinogen (mg/dL)	371.11 ± 162.05	346.78 ± 93.85	0.491
Total cholesterol (mg/dL)	179.10 ± 35.48	173.32 ± 35.57	0.525
Triglyceride (mg/dL)	143.13 ± 89.26	112.59 ± 48.96	0.110
HDL-cholesterol (mg/dL)	44.07 ± 15.61	45.0 ± 12.26	0.798
FBS (mg/dL)	107.92 ± 22.23	120.56 ± 62.71	0.347

^a^ *P* value using Student's *t*-test for continuous variables and chi-square test or Fisher's exact test ^*^for categorical variables.

**Table 2:** Distribution of BS symptoms in BS and non-BS patients.

Variable	Non-BS	BS	*P* value^a^
Black face with black eyelid	1 (3.2)	4 (12.9)	0.354^*^
Purpura in the sclera	5 (16.1)	2 (6.5)	0.425^*^
Purpura	6 (19.4)	9 (29.0)	0.554^*^
Cyanotic lips	3 (9.7)	10 (32.3)	0.059^*^
Site-fixed headache	2 (6.5)	5 (16.1)	0.425^*^
Headache with a pulling sensation	1 (3.2)	3 (9.7)	0.612^*^
Fishy-smelling mouth odor	15 (48.4)	11 (35.5)	0.440^*^
Tongue purple in color	2 (6.5)	13 (41.9)	0.001
Tongue with red, white, or black spots as well as thornlike protrusions on its surface	0 (0.0)	4 (12.9)	0.039
Bloated feeling in the chest and hypochondriac region	1 (3.2)	0 (0.0)	0.313^*^
Rough pulse	2 (6.5)	20 (64.5)	<0.001

^a^ *P* value using a chi-square test or Fisher's exact test (∗) for categorical variables.

**Table 3:** Identification of plasma metabolites in patients with cerebral infarction (non-BS and BS) analyzed using UPLC-Q-TOF MS.

Number^a^	Identity	Exact mass (M + H)	Actual mass (M + H)	Mass error (mDa)	MS fragments (ESI)	Fold change (BS versus non-BS)	*P* value^b^	VIP
1	Creatinine	114.0667	114.0670	−0.3	99, 86, 72, 71	1.46	0.010	2.88
2	Acetyl carnitine	204.1236	204.1262	−2.6	145, 144, 85, 60	1.44	0.037	1.75
3	Leucine	132.1025	132.1001	2.4	86	1.31	0.042	4.12
4	Kynurenine	209.0926	209.0998	−7.2	174, 146, 136, 94	2.22	0.010	1.05
5	Phosphocholine	185.0817	185.1326	−50.9	125, 98, 86	1.61	0.000	1.73
6	Hexanoyl carnitine	260.1862	260.1897	−3.5	201, 183, 144, 85	5.18	0.000	1.28
7	Decanoyl carnitine	316.2488	316.2512	−2.4	137, 129, 144, 85	1.98	0.012	1.42

^a^Number is the number of metabolites marked in Figure 1(c).

^b^ *P* value was calculated using Mann-Whitney *U* test.
