# Supplementary material for: Metabolomic Analysis of Clinical Plasma from Cerebral Infarction Patients Presenting with Blood Stasis
Source: Evid Based Complement Alternat Med. 2015 Mar 5;2015:453423. doi: 10.1155/2015/453423 (PMC4365334; doi:10.1155/2015/453423)
Supplement: Supplementary file 1 — Suppl. Table 1: Identification of plasma metabolites in stroke patients (Non-BS and BS) analyzed using ultra-performance liquid chromatography-quadrupole time-of-flight mass spectrometry. [file 453423.f1.pdf]

Suppl. Table 1. Identification of plasma metabolites in stroke patients (Non-BS and BS) analyzed using ultra-performance liquid chromatography-quadrupole time-of-flight mass spectrometry

Identity	Exact mass (M+H)	Actual mass (M+H)	Mass error (mDa)	MS fragments (ESI)	Fold change (BS vs. Non-BS)	P-value[a]	VIP
Proline	116.0712	116.0703	0.9	70	1.19	0.128	2.09
Valine	118.0868	118.0858	1.0	72	1.10	0.544	0.96
Betaine	118.0868	118.0873	-0.5	58	1.12	0.187	0.88
Nicotinamide	123.0558	123.0568	-1.0	123,80	1.29	0.833	0.33
Pyroglutamic acid	130.0504	130.0527	-2.3	84	1.11	0.228	0.54
Pipecolic acid	130.0868	130.0878	-1.0	91,84,72	2.02	0.186	1.36
Isoleucine	132.1025	132.1017	0.8	119,86,72	1.05	0.909	0.4
Aspartic acid	134.0453	134.0034	41.9	113,72	-1.18	0.646	0.49
Pheylacetamide	136.0762	136.0779	-1.7	119.107,91	1.13	0.384	0.63
Hypoxanthine	137.0463	137.0456	0.7	119.110.94.82	1.53	0.737	1.81
Glutamine	147.077	147.0807	-3.7	130,84	-1.04	0.863	0.15
Methionine	150.0589	150.0605	-1.6	130,116,70	1.35	0.077	1.5
Phenylglycine	152.0712	152.0724	-1.2	105,78	1.15	0.804	0.08
Carnitine	162.113	162.1146	-1.6	85,60	1.26	0.071	2.49
Hydrolysine	163.1083	163.1197	-11.4	128,82	1.08	0.586	0.15
Phenylalanine	166.0868	166.0832	3.6	120,103,93	1.11	0.149	2.93
Uric acid	169.0362	169.0367	-0.5	152,141,126,70	1.14	0.132	1.66

Arginine	175.1195	175.1241	-4.6	130,116,70	1.09	0.548	0.18
Tyrosine	182.0817	182.0831	-1.4	165,136,123	1.24	0.207	1.76
Gly-Leu	189.1239	189.1801	-56.2	171,143,86	-1.26	0.188	0.23
Tryptophane	205.0799	205.0951	-15.2	188,146,118	1.08	0.577	1.58
Isobutrylcarnitine	232.1549	232.1576	-2.7	217,144,85	1.31	0.364	0.50
Dodecanoylcarnitine	344.2801	344.2851	-5	285,144,85	-1.03	0.814	0.02
Linoeylcarnitine	424.3427	424.347	-4.3	352,144,85	-1.58	0.378	0.45
Vaccenylcarnitine	426.3583	426.3599	-1.6	367,144,85	1.00	0.696	0.00
LPC(C14:0)	468.309	468.3104	-1.4	450,184,104,86	-1.02	0.646	0.07
LPE(C18:2)	478.2934	478.295	-1.6	337,104	1.05	0.883	0.16
LPC(C15:0)	482.3168	482.327	-10.2	464,184,104,86	-1.08	0.784	0.13
LPC(C16:1)	494.3247	494.3245	0.2	476,184,104,86	-1.21	0.799	0.78
LPC(C16:0)	496.3403	496.337	3.3	478,184,104,86	-1.03	0.844	0.26
LPE(C20:4)	502.2934	502.2966	-3.2	361,104	1.22	0.383	0.52
LPC(C17:1)	508.3767	508.3784	-1.7	490,184,104,86	1.58	0.106	0.57
LPC(C17:0)	510.3556	510.3587	-3.1	492,184,104,86	-1.01	0.742	0.02
LPC(C18:3)	518.3247	518.3268	-2.1	500,184,104,86	-1.24	0.510	0.300
LPC(C18:2)	520.3403	520.3338	6.5	502,184,104,86	1.14	0.486	2.19
LPC(C18:1)	522.356	522.3499	6.1	504,184,104,86	-1.00	1.000	0.06
LPC(C18:0)	524.3716	524.3656	6	506,184,104,86	-1.08	0.838	1.53

LPC(C20:4)	544.3403	544.3396	0.7	526,184,104,86	1.07	0.664	0.48

[a], p value was calculated using Mann-Whitney U-test
